# Comparative Genomic Analysis of Type VII Secretion System in *Streptococcus agalactiae* Indicates Its Possible Sequence Type-Dependent Diversity

**DOI:** 10.3389/fcimb.2022.880943

**Published:** 2022-05-19

**Authors:** Kaixin Zhou, Lianyan Xie, Xiaogang Xu, Jingyong Sun

**Affiliations:** ^1^ Institute of Antibiotics, Huashan Hospital, Fudan University, Shanghai, China; ^2^ Department of Laboratory Medicine, Ruijin Hospital, Shanghai Jiaotong University School of Medicine, Shanghai, China; ^3^ Department of Clinical Microbiology, Ruijin Hospital, Shanghai Jiaotong University School of Medicine, Shanghai, China

**Keywords:** *S. agalactiae*, Type VII secretion system, sequence alignment analysis, sequence type (ST), insertion sequence

## Abstract

*Streptococcus agalactiae* causes sepsis and meningitis in neonates, presenting substantial clinical challenges. Type VII secretion system (T7SS), an important secretion system identified in *Mycobacterium* sp. and Gram-positive bacteria, was recently characterized in *S. agalactiae* and considered to contribute to its virulence and pathogenesis. In the present study, 128 complete genomic sequences of *S. agalactiae* were retrieved from GenBank to build a public dataset, and their sequences, capsular types, and clonal complexes were determined. Polymerase chain reaction (PCR) screening and genomic sequencing were conducted in an additional clinical dataset. STs and capsular types were determined using PCR. Eleven different types of T7SS were detected with similarities in gene order but differences in gene content. Strains with incomplete T7SS or lack of T7SS were also identified. Deletion, insertion, and segmentation of T7SS might be related to insertion sequences. The genetic environment of T7SS in *S. agalactiae* was also investigated and different patterns were identified downstream the T7SS, which were related to the diversity of T7SS putative effectors. The T7SS demonstrated possible sequence type (ST)-dependent diversity in both datasets. This work elucidated detailed genetic characteristics of T7SS and its genetic environment in *S. agalactiae* and further identified its possible ST-dependent diversity, which gave a clue of its mode of transmission. Further investigations are required to elucidate the underlying mechanisms and its functions.

## Introduction

*Streptococcus agalactiae* (Group B *Streptococcus*; GBS) is a Gram-positive bacterium that exists as a commensal in the gastrointestinal and genitourinary tracts of humans (Furfaro et al., 2018). However, this bacterium is a major cause of sepsis and meningitis in neonates and infants (Madrid et al., 2017) and an important pathogen in pregnant women, immunocompromised adults, and the elderly (Le Doare and Heath, 2013). The incidence of invasive GBS in nonpregnant adults has shown a continuous increase in recent years (Francois Watkins etal., 2019; Collin et al., 2020). Moreover, many animal species can be infected with GBS, with major economic impacts in the global dairy and aquaculture industries (Almeida et al., 2016; Delannoy et al., 2016; Richards et al., 2019; Crestani et al., 2021). It has also been shown to cause a foodborne disease associated with the consumption of raw fish in healthy adults in Singapore (Tan et al., 2016).

The first type VII secretion system (T7SS) was discovered in *Mycobacterium tuberculosis*, the main pathogen causing tuberculosis (Rivera-Calzada et al., 2021). The mycobacterial ESX secretion systems feature a set of five conserved core membrane components (EccB, EccC, EccD, EccE, and MycP), which mediate the secretion of the EsxA : EsxB family of virulence factors or DNA (Houben et al., 2012b; Gray et al., 2016; Famelis et al., 2019). ESX systems control diverse biological functions including host-pathogen interaction and inter-strain genetic transfer in mycobacteria. Five paralogous ESX loci (ESX-1 to ESX-5) are widespread among virulent and non-virulent mycobacteria (Rivera-Calzada et al., 2021). However, the five Mycobacterial ESX secretion systems perform distinct functions. For instance, ESX-1 is associated with virulence in *M. tuberculosis* (Samten etal., 2009; Houben et al., 2012a; Sreejit et al., 2014). In *Mycobacterium smegmatis*, a fast-growing and nonpathogenic species, ESX-1 and ESX-4 are involved in distributive conjugal transfer (Coros et al., 2008; Gray et al., 2016). In contrast, ESX-3 and ESX-5 is essential for mycobacterial growth (Sassetti et al., 2003; Siegrist et al., 2009; Ates etal., 2015).

The ESX gene clusters identified in Firmicutes (low G+C Gram-positive bacteria) (Gey Van Pittius et al., 2001; Abdallah et al., 2007) are evolutionary distant from those found in other bacteria; therefore, the subfamily was termed as T7SSb (Abdallah et al., 2007; Rivera-Calzada et al., 2021). The T7SSb apparatus comprises fewer protein subunits than Actinobacterial T7SSs. The T7SSb core machinery comprises four membrane proteins (EssA, EssB, EssC, and EsaA), constituting the main secretory machine in the bacterial cell envelope (Rivera-Calzada et al., 2021; Tran et al., 2021). *Staphylococcus aureus* was reported to have a functional T7SS that can secrete EsxA and EsxB (Burts et al., 2005), belonging to the WXG100 family of proteins, and EsxC and EsxD (Anderson et al., 2013), the two non-WXG100 proteins. Studies have shown that the mutants that fail to secret EsxA and EsxB display significantly reduced virulence, defective dissemination, and colonization (Burts et al., 2005). In addition, the virulent *Streptococcus suis*, an important zoonotic pathogen, secretes the EsxA protein that facilitates its invasion into mouse liver and kidneys (Lai et al., 2017). However, the putative T7SS of *Listeria monocytogenes* is not involved in virulence (Way and Wilson, 2005).

Recently, T7SS was also characterized in *S. agalactiae* and divided into at least four subtypes based on the C-terminus of EssC. It contributes to GBS pathogenesis mainly through allowing export of protein and/or toxins and promoting the disruption of blood-brain barrier (BBB). The secreted T7SS effector protein, EsxA, can form pores in lipid membranes. In a murine model of hematogenous meningitis, mice infected with GBS lacking a functional T7SS or lacking the secreted EsxA exhibited less mortality, lower bacterial burdens in tissues, and decreased inflammation in the brain. Deletion of the *essC* gene in the subtype I GBS strain mitigated GBS-induced inflammation in the brain and cell death in brain endothelial cells (Spencer et al., 2021).

T7SS has been well characterized in *Mycobacterium* sp. (Rivera-Calzada et al., 2021) and different types of ESX loci proved to control diverse biological functions. Therefore, it is necessary to elucidated detailed genetic characteristics of T7SS subtypes in *S. agalactiae* to providing a genetic basic for further functional investigations. Meanwhile, comparative genome analysis of *Staphylococcus lugdunensis* shows Clonal Complex (CC)-dependent diversity of T7SS (Lebeurre et al., 2019). However, it is unknown whether these phenomena also exist in *S. agalactiae*. This study elucidated the detailed genetic characteristics of the T7SS in *S. agalactiae* and further identify its sequence type (ST)-dependent diversity.

## Methods

### Bacterial Strains and Culture Conditions

A total of 131 nonduplicated GBS isolates were recovered during 2014–2021 at Ruijin Hospital, School of Medicine, Shanghai Jiao Tong University, Shanghai, China. Isolates were grouped into three different types based on the clinical outcomes and isolation sources: invasive isolates (n=3) defined as those derived from normally sterile body sites; non-invasive isolates (n=65) defined as those derived from non-sterile sites and with clinical infection diagnosis of the isolation site; colonizing isolates (n=63) defined as those derived from vaginal swabs and urine samples from asymptomatic patients (most maternal colonizing strains). Detailed data of the isolates is presented in **Supplementary Table 1**. All isolates were identified using MALDI-TOF MS (bioMérieux, Paris, France). All GBS isolates were grown in Todd-Hewitt broth (THB, BD) or Todd-Hewitt agar (THA) with 6% (v/v) sheep blood at 37°C in a CO_2_ incubator.

### Multilocus Sequence Typing Analysis and Serotyping

Genomic DNA was extracted using W izard^®^ Genomic DNA Purification Kit (Promega, Madison, WN, USA) according to the manufacturer’s protocol. MLST was performed as previously described (Jones et al., 2003). Briefly, seven housekeeping genes (*adhP, atr, glcK, glnA, pheS, sdhA*, and *tkt*) were amplified and sequenced. The *S. agalactiae* MLST database (http://pubmlst.org/sagalatiae; accessed on October 2, 2021) was used for assigning allele numbers and obtaining the sequence types (STs). Using the eBURST program, the STs were grouped into clonal complexes (CCs) whose members shared at least five of the seven MLST loci (Francisco et al., 2009); otherwise, the ST was considered a singleton. A multiplex polymerase chain reaction (PCR) assay was performed to identify the capsular type as previously described (Imperi et al., 2010). Briefly, a multiplex PCR was performed using a mix of 19 primers and each serotype was identified by the analysis of the unique two or three bands pattern on agarose gel.

### Public Data Set

A total of 128 complete *S. agalactiae* genomes were retrieved from the GenBank database at NCBI (https://www.ncbi.nlm.nih.gov/genome/browse#!/prokaryotes/streptococcus%20agalactiae; with a filter of assembly level as complete; accessed on October 6, 2021) to analyze the presence of the T7SS loci orthologs in *S. agalactiae* (**Supplementary Table 2**). Representative *S. aureus* strain COL (accession No.: NC_002951) and *Streptococcus. suis* strain GZ0565 (accession No.: CP017142) were also obtained and sequence alignment was conducted between the T7SS loci of these genomes by BLASTn and BLASTp. STs of all 128 strains were determined using the *S. agalactiae* MLST database (http://pubmlst.org/sagalatiae; accessed on October 6, 2021) and confirmed by a separate Web-based method on the basis of WGS data (www.cbs.dtu.dk/services/MLST) (Larsen et al., 2012). WGS-based identification of serotypes was conducted by employing the query DNA sequences and percent identity requirements as previously described (Metcalf et al., 2017) and confirmed using a serotyping database (https://github.com/swainechen/GBS-SBG) (Tiruvayipati et al., 2021).

### T7SS Detection

Comparative analysis and annotations of genome sequences were performed to detect T7SS between two clinical GBS strains Sag37 (accession No.: CP019978.1) and Sag158 (accession No.: CP019979.1) previously described (Zhou et al., 2017). The T7SS sequence in Sag37 (accession No.: CP019978.1; bases from 1,149,232 to 1,161,088) was used to identify the T7SS clusters in *S. agalactiae* through homology screening using the BLASTp and BLASTn against the NCBI database followed by careful manual inspection. Sequence analysis and comparison of genomes led to the identification of the related copies of T7SS. BLASTp and ClustalW were used to align and calculate the average amino acid sequence identity (Larkin et al., 2007).

### Phylogenetic Analysis of the Public Data Set

Assembled genomes in the public data set were mapped to a reference (Sag37) genome and single nucleotide polymorphisms (SNPs) were called using snippy v3.1 (https://github.com/tseemann/snippy). The phylogenetic tree was constructed using core SNPs alignment and visualized using the Interactive Tree of Life (iTOL) (https://itol.embl.de/) (Letunic and Bork, 2019).

### Amplification of T7SS-Associated Genes in Clinical Strains

PCR-based screening was performed to distinguish the different T7SS types and estimate their distribution in the clinical strains, as well as to determine the ST-dependent diversity observed in the isolates obtained from the GenBank database. The *essC-1, essC-2, essB, esaA*, and *esxA* genes were amplified to elucidate the general structure of the T7SS apparatus in the clinical strains. Different T7SS types were indicated by different PCR-based patterns. Specifically, the amplified sections of all five genes were present in Types A-D. Types E and F showed no amplification of *esxA* while the other four genes were identified; this was defined as pattern B. Type K was defined by the lack of amplification of all five genes. Types G and H were also distinguished through PCR amplification. Primers used in the present study were designed based on the conserved sequences of T7SS observed in the alignment of the publicly available genomes (**Supplementary Table 3**). DNA isolation, amplification, and electrophoresis of the PCR products were carried out as reported earlier (Hynes et al., 1992). The PCR products were analyzed by sequencing and BLASTp.

### Genome Sequencing, Assembly, Annotation

Genomic DNA of 17 representative clinical strains was extracted using Wizard^®^ Genomic DNA Purification Kit (Promega, Madison, WN, USA) according to the manufacturer’s protocol. Purified genomic DNA was quantified by a TBS-380 fluorometer (Turner Biosystems Inc., Sunnyvale, CA, USA). Next, approximately 1 μg genomic DNA was used from each strain to construct sequencing library. DNA samples were sheared into 400–500 bp fragments using a Covaris M220 Focused Acoustic Shearer (Trendbio, Melbourne, Australia) following the manufacturer’s protocol. Sequencing libraries were prepared from the sheared fragments using the NEXTflex™ Rapid DNA-Seq Kit (Tecan, Canton of Zürich, Switzerland). The prepared libraries were used for paired-end Illumina sequencing (2 × 150 bp) on an Illumina HiSeq X Ten machine (Illumina, San Diego, CA, USA). The original image data were transferred into sequence data *via* base calling, defined as raw data or raw reads and saved as a FASTQ file. The clean reads were assembled using the SOAPdenovo2 software (Koren et al., 2017). Glimmer (Delcher et al., 2007) was used for coding sequence (CDS) prediction, tRNA-scan-SE was used for tRNA prediction, and Barrnap was used for rRNA prediction. The predicted CDSs were annotated from NR, Swiss-Prot, Pfam, GO, COG and KEGG database using sequence alignment tools such as BLASTP, Diamond and HMMER. Furthermore, each set of query proteins were aligned with the databases, and annotations of best-matched subjects (e-value < 10-5) were obtained for gene annotation.

### Statistical Analysis

Statistical comparisons were performed *via* Fisher’s exact tests, as well as Chi-square and Cramer’s V analyses, considering values of P < 0.05 as statistically significant. Specifically, Fisher’s exact tests were performed to claim significant associations between T7SS subtypes and STs. Correlation between T7SS types and host range was determined by Fisher’s exact tests, as well as Chi-square and Cramer’s V analyses. All tests were performed using SAS software (version 9.4).

### Nucleotide Sequence Accession Numbers

The results of the whole-genome shotgun sequencing projects were deposited at DDBJ/ENA/GenBank under the corresponding accession numbers (**Supplementary Table 4**).

### Ethics Statement

This study was approved by the ethics committee of Ruijin Hospital, School of Medicine, Shanghai Jiao Tong University, Shanghai, China and the Review Board exempted the requirement for written informed consent because this is a retrospective study only focused on bacteria and did not affect the patients.

## Results

### T7SS Identification and Characterization in Clinical GBS Strain Sag37

**Figure 1:**
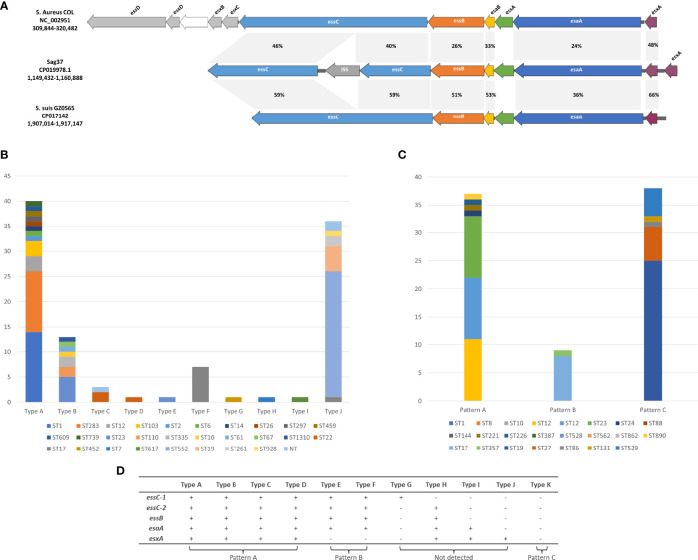
**(A)** Schematic representation of the T7SS gene clusters in *S. agalactiae* strain Sag37. Homology analysis and comparison against the corresponding clusters in different species including *Staphylococcus aureus* COL and *Streptococcus suis* GZ0565 is also indicated. Related genes are indicated by the same color and amino-acid identities for each gene are shown in the shaded grey areas. **(B)** Distribution of T7SS types in GBS isolates derived from the Genbank (Sag37 not included). NT: Non-typeable. **(C)** Distribution of different patterns of PCR screening in clinical GBS isolates recovered during 2014&ndash;2020. **(D)** Corresponding relationship between PCR-based patterns and T7SS subtypes retrieved from GenBank.

The T7SS apparatus was detected in the clinical GBS strain Sag37 isolated from the blood sample of neonate suffering from bacteremia (Zhou et al., 2017). The T7SS cluster was arranged in the order of *essC-essB- esaB-essA-esaA*-*esxA-esxA* (duplicated *esxA*), presenting insertion sequence IS5 in the *essC* gene (**Figure 1A**). Homology analysis demonstrated that the putative proteins encoded by the T7SS genes had limited similarity with those found in the *S. aureus* strain COL and *S. suis* strain GZ0565 (**Figure 1A**). The amino-acid identities of the proteins encoded by the T7SS genes varied between 36&ndash;59% in Sag37 vs. *S. suis* GZ0565 and between 24&ndash;46% in Sag37 vs. *S. aureus* COL; however, no significant similarity was observed in *essA* between the strains. *Staphylococcus aureus* possessed a more complicated T7SS apparatus than *S.* species, with eleven type VII system-associated genes (Burts et al., 2005; Anderson et al., 2013). The *essD, esxD, esxB*, and *esxC* genes found in the *S. aureus* strain were absent in *S. agalactiae* and *S. suis*.

### Public Data Set

A total of 128 complete *S. agalactiae* genomes were retrieved from the GenBank database to build a public data set and investigate the T7SS gene cluster and its genetic environment in *S. agalactiae*. Thirty STs were included and arranged into six major CCs groups (CC1, CC7, CC10, CC19, CC61, and CC260) except for three non-typeable strains as only six housekeeping genes were identified (**Table 1**). CC260 was the most predominant group (30.4%), followed by CC1 (13.3%) and CC19 (7.8%) (**Table 2**). Furthermore, 43 isolates (33.6%) were grouped as singletons. Serotypes of 128 strains included Ia, Ib, II, III, IV, V, VI, and VII. In addition, four non-typeable isolates were identified. Serotype Ib was the most predominant (39.1%), followed by III (23.4%) and V (13.3%), respectively. 62 strains were animal-derived with the majority of ST260/261/283 (mainly fish), while 56 strains were human-derived with the majority of ST1/12/17/19/23/283.

**Table 1:** Sequence types (STs) grouped by clonal complex (CC) for GBS isolates recovered from GenBank and clinical GBS isolates recovered during 2014–2020.

Clonal Complex	STs for isolates from Genbank (No. isolates)	STs for clinical isolates (No. isolates)
CC1	ST1 (14); ST2(1); ST14 (1); ST297 (1)	ST1 (11); ST387 (2)
CC7	ST6 (1); ST7 (7)	/
CC10	ST10 (2); ST12 (4)	ST10 (22); ST12 (11); ST8 (1); ST562 (2)
CC17	/	ST17 (8); ST357 (1)
CC19	ST19 (5); ST110 (2); ST335 (2), ST928 (1)	ST19 (25); ST27 (6); ST86 (1); ST131 (1)
CC23	/	ST23 (11); ST24 (15); ST88 (1); ST144 (1); ST221 (1); ST528 (1); ST890 (1)
CC61	ST61 (1); ST67 (1)	/
CC260	ST260 (9); ST552 (25); ST927 (5)	/
Singletons	ST17 (8); ST22 (3); ST23 (5); ST26 (1); ST103 (3); ST261 (5); ST283 (12); ST452 (1); ST459 (1); ST609(1); ST617 (1); ST739 (1); ST1310 (1)	ST529 (5); ST862 (3); ST226 (1)
NT^a^	3	/

a NT, Non-typeable.

**Table 2:** Clonal complex (CC) typing and capsular type of GBS isolates recovered from GenBank and clinical GBS isolates recovered during 2014–2020.

	Capsular type^a^	Ia	Ib	II	III	IV	V	VI	VII	NT^b^	Total
GenBank	CC1	0	0	0	0	0	13	3	1	0	17 (13.3%)
	CC7	7	1	0	0	0	0	0	0	0	8 (6.3%)
	CC10	0	4	1	0	1	0	0	0	0	6 (4.7%)
	CC19	0	0	0	8	0	2	0	0	0	10 (7.8%)
	CC61	0	0	2	0	0	0	0	0	0	2 (1.6%)
	CC260	0	38	0	0	0	0	0	0	1	39 (30.4%)
	Singletons^c^	5	5	6	20	2	2	1	0	2	43 (33.6%)
	NT^b^	0	2	0	0	0	0	0	0	1	3 (2.3%)
	Total	12 (9.4%)	50 (39.1%)	7 (5.5%)	30 (23.4%)	3 (2.3%)	17 (13.3%)	4 (3.1%)	1 (0.8%)	4 (3.1%)	128 (100%)
Clinical isolates	CC23	28	0	0	0	0	3	0	0	0	31 (23.7%)
	CC10	1	31	3	0	0	1	0	0	0	36 (27.5%)
	CC19	0	0	0	27	0	6	0	0	0	33 (25.2%)
	CC17	0	0	0	9	0	0	0	0	0	9 (6.8%)
	CC1	0	0	1	0	12	0	0	0	0	13 (9.9%)
	Singletons^d^	0	0	0	4	0	5	0	0	0	9 (6.9%)
	Total	29 (22.1%)	31 (23.7%)	4 (3.1%)	40 (30.5%)	12 (9.2%)	15 (11.4%)	0 (0%)	0 (0%)	0 (0%)	131 (100%)

a Not including three non-typeable strains.

b NT, non-typeable.

c Including ST17 (III: 7/NT:1); ST22 (II: 3); ST23 (Ia:2/III:2/NT: 1); ST26 (V: 1); ST103 (Ia: 3); ST261 (Ib: 5); ST283 (III: 12); ST452 (IV: 1); ST459 (IV: 1); ST609 (V: 1); ST617 (VI: 1); ST739 (III: 1); ST1310 (II: 1).

d Including 1 ST226 (type III), 5 ST529 (type V), 3 ST862 (type III).

### Characterization of T7SS in Public Data Set

**Figure 2:**
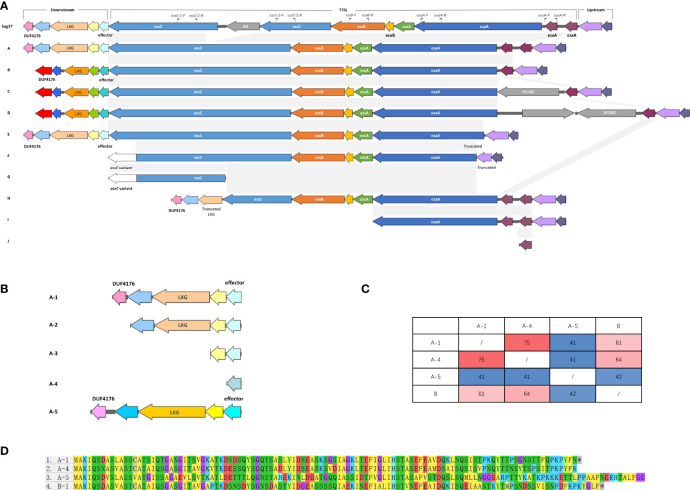
**(A)** Schematic representation of the T7SS gene clusters and their flanking genes, both upstream and downstream. In the downstream flanking genes, genes encoding similar conserved domains/motifs are indicated with similar colors; genes in pink and light purple encode DUF4176 domain-containing proteins; genes in beige and orange encode LXG domain-containing proteins; genes in cyan and turquoise encode T7SS putative effector. In the T7SS genes, genes with coverage and percent identity greater than 99% are indicated by the same color and shaded in grey. Insertion sequences are colored in grey, as well as the gene annotated as hypothetical in Type D Downstream flanking genes displayed few differences, except for a deletion in Type F Identical genes are indicated by the same colors. Truncated genes are indicated. Type K, entirely lacking T7SS, is not shown. Locations of the primers used in this study are indicated by grey flags. **(B)** Schematic representation of five sub-patterns of the upstream sequences in Type A lacking DUF4176 domain-containing protein and/or LXG domain-containing protein. Genes encoding similar conserved domains/motifs are indicated with similar colors; gene in pink and light purple encodes DUF4176 domain-containing protein; genes in beige and orange encodes LXG domain-containing protein; gene in cyan, grey-blue and blue encodes T7SS putative effector. **(C)** Mutual percent identities between the different T7SS putative effectors. **(D)** ClustalW alignments of T7SS putative effector-encoding genes.

The screening for the T7SS coding regions within *S. agalactiae* strains demonstrated that T7SS was harbored on chromosomes and not on plasmids. Screening yielded eleven different types of genetic characteristics of T7SS that displayed similarities in gene order but differences in gene content (**Figure 2A**). The clusters with coverage and percent identity greater than 99% were grouped into the same type.

T7SS in Sag37 shared significant similarity with Type A except for the disruption of insertion sequence IS5 in *essC*. Type B had only one *esxA*, while Sag37 and type A had two tightly duplicated *esxA*. Type C was similar to Type B except for an IS1182 family transposase locating downstream of the *esxA*. Compared with Type C, Type D had an extra hypothetical protein downstream the IS1182 family transposase. Incomplete T7SS clusters with obvious deletions were also observed during comparison. A ~300 bp deletion at the end of the *esaA* was observed in Type E, with deletion of subsequent *esxA* as well. Type F had more deletion (~550 bp deletion) up to the flanking gene downstream. Distinct *essC* variants was detected in Type F and G with a ~650 bp difference in the C-terminus from the aforementioned types. Truncated T7SS was also observed. Incidentally, strains completely lacking T7SS were also discovered (Type K, not shown in **Figure 2A**).

Among all the types analyzed by us (Sag37 not included), type A formed the majority (36.2%), followed by Type K with 29.1% (**Figure 1B**). The proportion of both Type B and J exceeded 10%, whereas Type D/E/G/H/I appeared in only one representative strain in our data set.

### Flanking Fragments of T7SS

Putative T7SS effector-encoding genes were detected downstream *essC*, together with genes encoding DUF4176 domain-containing proteins and LXG domain-containing proteins. Although the proteins encoded in this region contained similar conserved domains/motifs, the downstream flanking nucleotide sequences were of distinct patterns. Types A and E were of the same pattern, while Types B, C, and D were of another. Aside from the major patterns illustrated in **Figure 2A**, five subpatterns were observed with the lack of genes coding for DUF4176 domain-containing proteins and/or LXG domain-containing proteins (**Figure 2B**). Moreover, a distinct A-5 pattern was observed with no homology to other patterns. Four different T7SS effector-encoding genes were detected in the public dataset. Although no significant similarity was found in the nucleotide sequences between A-5 and A-1, similar conserved domains/motifs were encoded in this region. Percent identities between the T7SS effector among the different subtypes were within 41-75% (**Figures 2C, D**). Similar structures were absent in types with *essC* variants (Type E and F), and the T7SS effector-encoding gene was absent in Type H. Compared to the diversified downstream flanking genes, upstream flanking genes displayed few differences.

### T7SS Apparatus in Clinical GBS Strains

Among 131 clinical GBS strains analyzed in this study, 22 STs were included and grouped into five major CCs (CC1, CC10, CC17, CC19, and CC23) (**Table 1**). CC10, CC19, and CC23 account for 76.4% of strains (**Table 2**). Nine isolates (singletons) including ST529, ST862, and ST226 were not related to any of the five CCs. Serotypes of all the 131 strains included Ia, Ib, II, III, IV, and V. Serotypes III was the most predominant (30.5%), followed by Ia (22.1%), and Ib (23.7%).

The PCR screening of the clinical strains demonstrated three patterns of T7SS clusters (see **Figure 1C**). Strains with identical STs showed similar results. Specifically, 84 isolates (64.1%) harbored all five genes present in Types A-D (see **Figure 1D**, Pattern A). Thirty-eight isolates (29.0%) harbored none of these genes (see **Figure 1D**, Pattern C). Nine isolates (6.9%) displayed pattern B. Patterns indicating Types G and H were not detected through PCR amplification.

The specific number of *esxA* genes could not be determined due to the limitation of the PCR. Hence, we performed genome sequencing; 17 strains presenting different patterns were selected and sequenced (see **Supplementary Table 4**). Further analysis revealed four T7SS types similar to that of the strains obtained from the GenBank database; strain 189 (ST10), which displayed type A T7SS rather than type C or E found in the GenBank, was the exception.

### Relationship Between ST and T7SS Characterization

**Figure 3:**
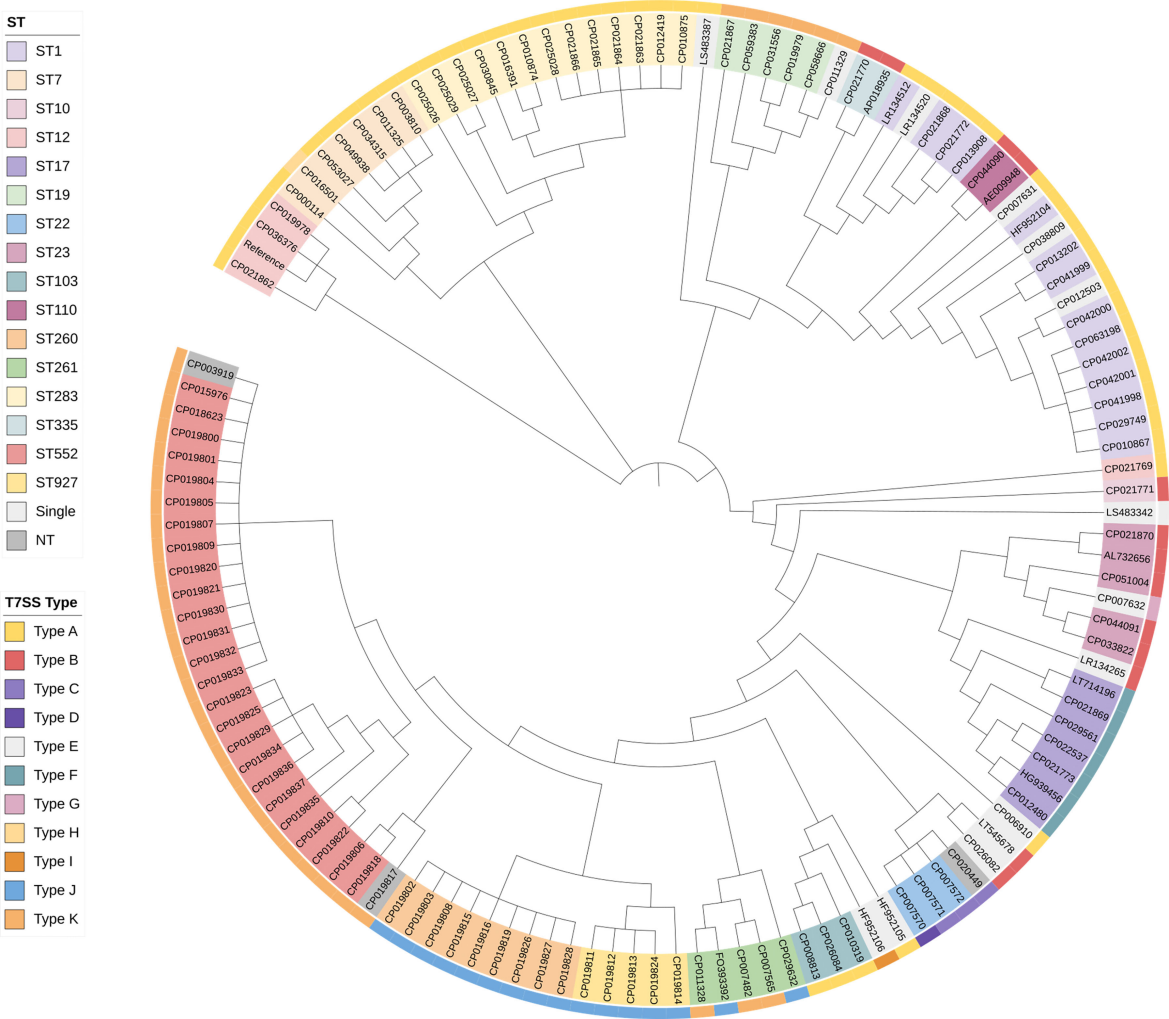
Phylogenetic relationship of 128 complete *S. agalactiae* genomes in the public dataset. Strains with identical STs are indicated by the same color in the phylogenetic tree. The outer ring indicates different T7SS types characterized in this study. Color coding is defined in the inset. Single, STs represented by a single genome in the database; NT, Non-typeable.

Characterization of the T7SS gene clusters showed a possible relation to the STs. Strains with identical STs in the public dataset, collected in different study covering a wide geographical range and time span, were found that could be grouped into the same T7SS types. The phylogenetic tree constructed based on the core SNPs alignment further confirmed that genetically closely associated strains had the same type of T7SS system (**Figure 3**). Exceptions were also found. Therefore, Fisher&rsquo;s exact tests were performed to identify correlations. Some of the STs were represented by a single genome in the database, which could only bear a single T7SS type. Therefore, STs that appeared only once were excluded from comparisons. Significant associations were found between most strains and types displayed in **Figure 2A**, including Type A with ST1/7/103/283, Type B with ST23/110/335, Type F with ST17, Type J with ST260/927, and Type K with ST19/552. For some of the less represented STs carrying multiple T7SS types, e.g., ST10, ST12, ST22, ST261, no correlations were found. No significant correlations were found between CCs/serotypes and T7SS characterization.

In our public dataset, 56 strains were human-derived while 62 strains were animal-derived. Correlation between T7SS types and host range was identified by Fisher&rsquo;s exact tests. To further evaluate the relationships between T7SS types and host range, Chi-square and Cramer&rsquo;s V analyses were performed between the T7SS subtypes discovered in both origins. T7SS subtypes demonstrated strong positive correlations with the host range (p &lt; 0.05, Cramer&rsquo;s V=0.538). Statistical significance was claimed by adjusted residuals that human-derived GBS was associated with complete T7SS apparatus (Type A and B), whereas animal-derived GBS with an incomplete one (Type K).

## Discussion

T7SS has been well characterized in *Mycobacterium* species, in terms of its structure, functions, and transport models (Rivera-Calzada et al., 2021). Recent advances have also facilitated our understanding of the T7SS apparatus in *S. agalactiae* (Spencer et al., 2021). In the present study, the T7SS apparatus was detected in clinical GBS strain Sag37 isolated from blood samples of neonates suffering from bacteremia (Zhou et al., 2017). We elucidated the genetic characteristics of the T7SS in *S. agalactiae* in detail and further identified its ST-dependent diversity. Strains with incomplete T7SS or lack of T7SS were also identified. The genetic environment of T7SS in this species was investigated and different patterns were further identified downstream of the T7SS, which was related to the diversity of T7SS putative effectors.

A total of 128 complete *S. agalactiae* genomes were collected and a public database was built. Multiple T7SS types were distinguished during subsequent comparisons, and strains with incomplete T7SS or lack of T7SS were discovered. Distinct *essC* variants were detected in Types F and G with a ~650 bp difference in the C-terminus, which was also regarded as the basis of the classification described by Spencer etal. (Spencer et al., 2021). In the incomplete Type H, genes encoding LXG domain-containing proteins were truncated and T7SS effector-encoding genes were not present, as well as the first half of *essC*. The truncation point was at the position where the IS5 family transposase was inserted in the T7SS of Sag37. The *essC* in Type G was also truncated at the same position and no sequences were found afterwards IS5. Moreover, compared to Type B, an IS1182 family transposase was embedded upstream *esxA* in Type C, while Type D had an extra hypothetical protein upstream the IS1182 family transposase. These results indicated that deletion, insertion, and segmentation of T7SS might be related to the insertion sequences.

In the public dataset, T7SS demonstrated ST-dependent diversity in *S. agalactiae*. Strains with identical STs tended to have similar T7SS types. The phylogenetic tree further confirmed that genetically closely associated strains commonly had the same type of T7SS system. However, some STs and types were limited and less represented, and thus reliable correlations could not be performed. To validate the correlations, a database of 131 clinical GBS strains was established, and PCR screening and genomic sequencing were used. Significant associations between the vast majority of strains and types were found by Fisher&rsquo;s exact tests. Exceptions were attributed to insertion sequences. For instance, an extra IS5 was embedded in T7SS of Sag37 compared to other strains of ST12 (Type A). This phenomenon also exists in *Staphylococcus lugdunensis* with CC-dependent diversity (Lebeurre et al., 2019). However, no significant correlations were found between CCs/serotypes and T7SS characterization in the present study.

ESX gene clusters located on plasmids have been reported in several mycobacterial species. Phylogenetic analysis has suggested that ESX-encoding plasmids are a major driving force for the acquisition and diversification of type VII systems in mycobacteria (Dumas et al., 2016; Newton-Foot et al., 2016). Based on the ST-dependent diversity found in *Streptococcus agalactiae*, it was hypothesized that T7SS was more likely to spread clonally rather than horizontally within this species. Only chromosomal-origin T7SS was detected in our screening of the two datasets of *S. agalactiae*. However, conclusions could not be drawn due to insufficient plasmids and limited host range (most human-derived) in the datasets. The T7SS genes encoding the putative proteins in Sag37 were homologous to those found in *S. aureus* and *S. suis* strains, although the similarity was limited. This indicated the possibility of genetic exchange between genera. Further investigations are required to validate our findings and elucidate the underlying mechanism.

Spencer etal. have characterized four example strains across the GBS T7SS subtypes based on the C-terminus of EssC (Spencer etal., 2021). The detailed analysis of the genetic environment of T7SS conducted in the present study further identified different patterns upstream the T7SS. Some of these patterns lacked the genes encoding DUF4176 domain-containing proteins and/or LXG domain-containing proteins, and even the deletion of T7SS putative effector-encoding genes. It appeared pattern variability depended on the diversity of the putative T7SS effector-encoding genes adjacent to *essC*

Identified T7SSs have been shown to secrete the WXG100 proteins involved in the virulence of some Actinobacteria and Firmicutes (Pym et al., 2003; Stanley et al., 2003; Burts et al., 2005; Anderson et al., 2013; Lai et al., 2017). Recently, the role of T7SS in GBS pathogenesis has also been assessed. GBS lacking a functional T7SS or lacking the secreted WXG100 effector EsxA exhibited reduced virulence and pathogenicity (Spencer et al., 2021). It is worth noting that statistical significance was claimed that human-derived GBS was associated with complete T7SS apparatus (Type A and B), whereas animal-derived GBS with an incomplete one (Type K). However, correlation between STs and T7SS types cannot be excluded.

In conclusion, T7SS in *S. agalactiae* demonstrated various genetic characteristics and ST-dependent diversity. Strains with incomplete T7SS or lack of T7SS were also identified. Different patterns were further identified downstream of the T7SS, which was related to the diversity of T7SS putative effectors.

## Data Availability Statement

The datasets presented in this study can be found in online repositories. The names of the repository/repositories and accession number(s) can be found in the article/**Supplementary Material**.

## Ethics Statement

The studies involving human participants were reviewed and approved by the ethics committee of Ruijin Hospital, School of Medicine, Shanghai Jiao Tong University, Shanghai, China. Written informed consent for participation was not required for this study in accordance with the national legislation and the institutional requirements.

## Author Contributions

All authors listed have made a substantial, direct and intellectual contribution to the work, and approved it for publication.

## Funding

This work was sponsored by the National Key Research and Development Program of China (Grant No. 2018YFE0101800), the Natural Science Foundation of Shanghai (Grant No. 20ZR1433900), the National Natural Science Foundation of China (Grant No. 81902116), and Shanghai Sailing Program (Grant No. 19YF1431400).

## Conflict of Interest

The authors declare that the research was conducted in the absence of any commercial or financial relationships that could be construed as a potential conflict of interest.

## Publisher’s Note

All claims expressed in this article are solely those of the authors and do not necessarily represent those of their affiliated organizations, or those of the publisher, the editors and the reviewers. Any product that may be evaluated in this article, or claim that may be made by its manufacturer, is not guaranteed or endorsed by the publisher.

## Supplementary Material

Supplementary Table 1 Clinical isolates.

Supplementary Table 2 Public data set.
