# Supplementary material for: Comparative Genomic Analysis of Type VII Secretion System in Streptococcus agalactiae Indicates Its Possible Sequence Type-Dependent Diversity
Source: Front Cell Infect Microbiol. 2022 May 19;12:880943. doi: 10.3389/fcimb.2022.880943 (PMC9160427; doi:10.3389/fcimb.2022.880943)
Supplement: Supplementary file 3 [file Table_3.docx]

**Supplementary Table 3.** Primers used in this study

| Gene | Primer | Sequence (5′-3′) | Product size (bp) |
| --- | --- | --- | --- |
| *essC-1* | essC-1-F | TAGAGGTGGTAGCCACGGTT | 737 |
|  | essC-1-R | TCTTCAAGGACCTGCCACAC |  |
| *essC-2* | essC-2-F | AACCAACGCATCCAAGACCA | 766 |
|  | essC-2-R | ATTTTCACCGCTCACCTCGT |  |
| *essB* | essB-F | AGGCCAACTCGTACTTCTGTG | 653 |
|  | essB-R | AGCGCACGCTCTATCTTTTG |  |
| *esaA* | esaA-F | GAAGGCCACTAACAGTCGCT | 916 |
|  | esaA-R | AGCCAAGATGAATTGGCGGA |  |
| *esxA* | esxA-F  esxA-R | TCTGCATCCGTCTGCTCAAT  ACACCAGAAGAACTTCGCTCA | 248 |

**Supplementary Table 4**. Accession numbers of GBS isolates obtained in this study

| Isolate | CC | ST | T7SS type | Accession Number |
| --- | --- | --- | --- | --- |
| ZDN52 | 1 | 1 | A | JAJGRR000000000 |
| 189 | 10 | 10 | A | JAJGRK000000000 |
| 167 | 10 | 12 | A | JAJGRI000000000 |
| B2 | 10 | 12 | A | JAJGRL000000000 |
| B3 | 10 | 12 | A | JAJGRM000000000 |
| 149 | 10 | 12 | A | JAJGRH000000000 |
| 179 | 23 | 23 | B | JAJHSW000000000 |
| ZDN34 | 23 | 23 | B | JAJGRP000000000 |
| 171 | 17 | 17 | F | JAJGRJ000000000 |
| F11 | 17 | 17 | F | JAJHSX000000000 |
| F11-M | 17 | 17 | F | JAJGRO000000000 |
| ZDN51 | 17 | 17 | F | JAJGRQ000000000 |
| bd-2 | 19 | 19 | K | JAJGRN000000000 |
| ZDN62 | 19 | 19 | K | JAJGRS000000000 |
| ZDN63 | 19 | 19 | K | JAJGRT000000000 |
| ZDN65 | 19 | 19 | K | JAJGRU000000000 |
| ZDN66 | 19 | 27 | K | JAJGRV000000000 |
